# AQbD-Driven RP-HPLC Method for Rapid Dissolution Monitoring of Amiodarone Hydrochloride Tablets Without Sample Dilution

**DOI:** 10.3390/ph19081255

**Published:** 2026-08-09

**Authors:** Ju-Hyun Yoon, Joo-Eun Kim

**Affiliations:** 1 Department of Biopharmaceutical Chemistry, Kookmin University, Seoul 02707, Republic of Korea; 2 Biopharmaceutical Chemistry Major, School of Applied Chemistry, Kookmin University, Seoul 02707, Republic of Korea; 3 Department of Pharmaceutical Engineering, Kookmin University, Seoul 02707, Republic of Korea

**Keywords:** high-performance liquid chromatography, dissolution, polysorbate 80, amiodarone hydrochloride, sample dilution, method validation

## Abstract

**Background/Objectives**: In this study, a novel reversed-phase high-performance liquid chromatography (RP-HPLC) method was established for the quality control of amiodarone hydrochloride, overcoming the limitations of the legacy compendial protocol. The conventional United States Pharmacopeia method requires a 10-fold sample dilution to avoid column overload and matrix interference from polysorbate 80. Attempting direct injection under conventional conditions severely compromises data reliability, leading to severe band broadening and peak distortion. **Methods**: To fairly evaluate the intrinsic separation capacity of the analytical systems, preliminary experiments were conducted using identical, undiluted dissolution samples. Through precise chromatographic redesign, the novel method eliminates the pre-treatment dilution step. Furthermore, the reliability, precision, and robustness of the proposed method were comprehensively validated in strict accordance with the International Council for Harmonisation Q2 guidelines. **Results**: Even upon direct injection of highly concentrated samples, the optimized system focuses broad peaks into sharp, symmetric, and narrow bands, increasing analytical sensitivity and theoretical plates. Furthermore, it maximizes intra-column separation efficiency, achieving complete baseline isolation of amiodarone from surfactant interferences within a retention time of approximately 6.6 min, thereby significantly improving analytical throughput. **Conclusions**: By eliminating cumbersome sample preparation, minimizing analysis time, and enhancing quantitative integrity, this validated RP-HPLC method offers an efficient, high-throughput standard analytical platform. It is highly suitable for rapid routine quality control and high-precision dissolution testing of generic amiodarone pharmaceuticals in industrial settings.

## 1. Introduction

Amiodarone hydrochloride is a potent antiarrhythmic agent widely prescribed for the treatment of ventricular and atrial arrhythmias [1,2]. Classified as a Class III agent under the Vaughan Williams classification system, this drug primarily suppresses arrhythmias by blocking potassium (K^+^) channels in myocardial cells, thereby delaying repolarization and prolonging the refractory period [3,4,5]. Notably, amiodarone exhibits multifaceted pharmacological properties that are not confined to a single mechanism; it simultaneously inhibits sodium (Na^+^) and calcium (Ca^2+^) channels and exerts beta-adrenergic receptor antagonism, effectively acting on all phases of the cardiac action potential [6,7,8,9]. For a drug with such potent pharmacological activity, precise control over its dissolution profile—which represents a critical stage for systemic drug absorption—is of paramount importance to guarantee patient safety and therapeutic efficacy. Consequently, the establishment of a highly reliable analytical method to monitor the dissolution behavior of amiodarone and ensure robust data reproducibility during routine quality control phases is essential [10,11,12]. The United States Pharmacopeia (USP) monograph for amiodarone tablets provides multiple dissolution test methods to accommodate various formulations. Among these, our study deliberately focuses on the method employing polysorbate 80 in the dissolution medium coupled with a high-performance liquid chromatography analytical finish. Because amiodarone is a highly lipophilic compound, the incorporation of a surfactant is frequently essential to ensure adequate solubility and maintain sink conditions during in vitro testing. While our previous study [10] established an optimized reversed-phase high-performance liquid chromatography (RP-HPLC) method for the assay (content uniformity) of amiodarone by overcoming matrix effects from tablet excipients, dissolution monitoring presents a fundamentally different and more complex analytical challenge. Unlike assay analysis, the dissolution medium for amiodarone requires a high concentration of surfactant (polysorbate 80) to facilitate drug solubility. This complex matrix introduces severe chromatographic complications.

The current USP method for amiodarone tablets stipulates a pre-treatment step involving a 10-fold sample dilution prior to analysis to avoid interference from highly concentrated samples and dissolution medium components. However, in this study, to fairly compare only the intrinsic separation capacity of the two methods, the chromatographic performance was evaluated by applying the legacy method under the same undiluted sample conditions. Consequently, the introduction of the undiluted sample matrix induced severe column overload and interference with polysorbate 80, and a significant decrease in the symmetry and resolution of the principal peak was observed [13,14]. These outcomes significantly deviated from conventional system suitability criteria; in particular, the severe peak fronting and band broadening were identified as critical factors compromising the accuracy and reproducibility of the quantitative analysis. Furthermore, the excessively long retention time of the amiodarone peak reduced the efficiency of the analytical cycle, and the 10-fold dilution pre-treatment process for every analysis introduces additional processing time in routine industrial quality control (QC) environments that require the rapid, high-throughput processing of large batches of dissolution samples [15,16].

To overcome these limitations of the legacy method, this study departed from the traditional trial-and-error approach and comprehensively adopted the Analytical Quality by Design (AQbD) methodology in alignment with the latest International Council for Harmonisation (ICH) guidelines [17,18,19]. First, an analytical target profile (ATP) defining the ultimate objective of the method was established, and peak resolution, symmetry, tailing factor, theoretical plate number, and analysis time were defined as critical analytical attributes (CAAs) governing data quality. Subsequently, five critical variables crucial to system suitability—namely, organic modifier ratio, mobile phase pH, flow rate, column temperature, and buffer concentration—were scientifically identified through preliminary hazard analysis (PHA) and failure mode and effects analysis (FMEA) [20,21,22]. Based on the risk assessment outcomes, these critical method parameters were systematically controlled. As a result, an optimal mobile phase pH condition was established to suppress the activity of residual silanol groups and prevent peak distortion, along with an organic solvent composition that eliminated interference from the surfactant (polysorbate 80). Furthermore, by increasing the proportion of acetonitrile to enhance the eluotropic strength and precisely modulate hydrophobic interactions, a reduction in analysis time and improved peak symmetry were achieved simultaneously. This was accomplished even when highly concentrated dissolution samples were directly injected without the 10-fold pre-dilution step, thereby maximizing laboratory analytical throughput and sample preparation efficiency without inducing column overload [23,24].

Finally, to validate the scientific reliability of the proposed optimized RP-HPLC method, a comprehensive method validation was conducted in strict compliance with the ICH guidelines [25,26]. The validation parameters encompassed specificity—demonstrating the clear isolation of the target analyte from the complex dissolution matrix—along with linearity, accuracy, precision, and robustness. Additionally, the limit of detection (LOD) and limit of quantitation (LOQ) were established to ensure precise quantification even in low-concentration dissolution samples. Through this systematic verification, the novel analytical method established in this study was confirmed to possess the optimal analytical integrity required for a robust dissolution monitoring and quality assessment tool for amiodarone formulations.

## 2. Results and Discussion

### 2.1. Method Development and Optimization

#### 2.1.1. Assessment of the Legacy Compendial Method

**Figure 1:**
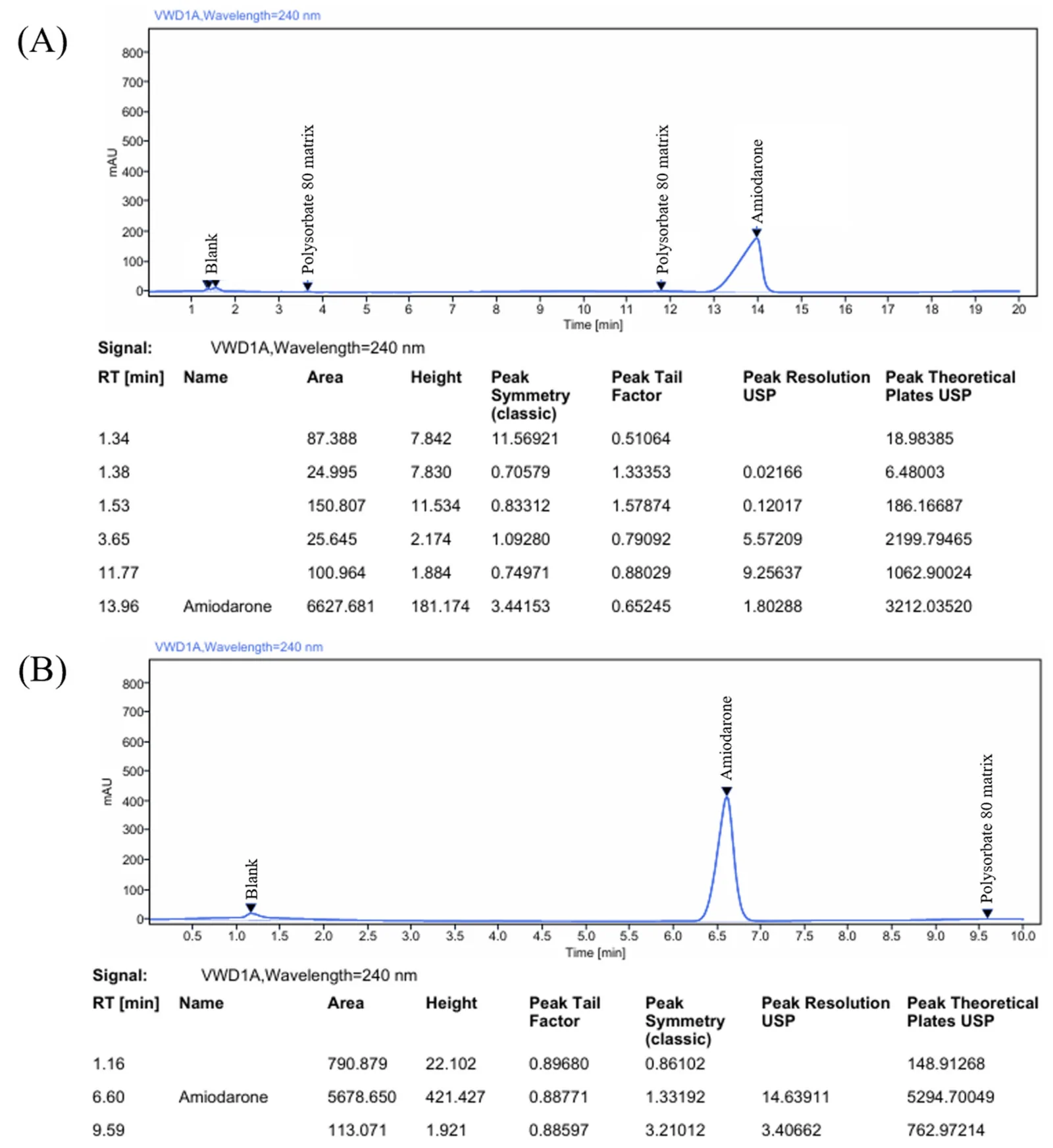
Chromatograms of amiodarone obtained from (**A**) the legacy method and (**B**) the optimized RP-HPLC method.

The development of the RP-HPLC method for the efficient analysis of amiodarone dissolution samples was conducted by carefully considering the physicochemical properties of the analyte, including its strong hydrophobicity and its interaction with the surfactant (polysorbate 80) present in the dissolution medium. Due to its high lipophilicity, amiodarone tends to exhibit excessive retention or severe peak fronting under conventional reversed-phase chromatographic conditions, presenting an analytical challenge in achieving clear isolation from the complex components derived from the dissolution matrix. According to the current USP amiodarone monograph, a 10-fold sample dilution prior to injection is recommended to prevent such column overload. However, to fairly evaluate only the intrinsic separation capacity of the newly developed method under high-throughput processing conditions, preliminary experiments were conducted using undiluted dissolution samples for both the legacy and optimized methods. Consequently, as illustrated in Figure 1A, the legacy USP-based method could not accommodate the undiluted sample, resulting in severe band broadening and peak asymmetry due to column overload and matrix interference from polysorbate 80. Furthermore, excessive column retention delayed the overall analysis time to nearly 20 min.

#### 2.1.2. Stepwise Optimization of the Separation Conditions

To overcome the limitations of the legacy method and maximize analytical efficiency, the selection of the appropriate separation conditions was systematically conducted in three progressive stages. Stage 1 involved evaluating the baseline legacy conditions, which, as previously noted, resulted in severe band broadening and an excessive analysis time due to matrix interference. Stage 2 focused on resolving the severe peak distortion by screening various buffer systems. Near-neutral pH caused partial ionization and severe peak distortion. Therefore, an acidic buffer (pH 4.0) supplemented with triethylamine (TEA) was ultimately selected. The acidic environment ensures uniform ionization of amiodarone, while TEA acts as an effective silanol-blocking agent. This optimization effectively blocked secondary interactions with the residual silanol groups of the silica-based stationary phase, thereby drastically improving peak symmetry. Once peak symmetry was secured, Stage 3 involved precisely controlling the eluotropic strength to achieve a rapid yet clean separation from the dissolution matrix. This was accomplished by systematically titrating the blending ratio of the organic modifiers (methanol and acetonitrile). Initial trials utilizing solely methanol resulted in excessively long retention times, whereas utilizing solely acetonitrile caused the amiodarone peak to elute too quickly, leading to co-elution with the massive polysorbate 80 matrix peak. By finely adjusting the proportion of both solvents, an optimal balance was achieved that completely isolated the target peak from the surfactant interference while drastically shortening the run time. Representative chromatograms illustrating these intermediate developmental stages and the progressive improvement in peak shape and resolution are provided in Figure S1.

#### 2.1.3. Practical Advantages and Sustainable Application of the Final Method

The final chromatographic conditions were established with a primary focus on maximizing the speed and reproducibility of the analytical cycle, rendering it highly suitable for routine QC testing and the high-throughput processing of large dissolution sample batches. As a result, the optimized method (Figure 1B) successfully achieved a sharp, highly symmetric peak with excellent resolution at a retention time of approximately 6.6 min, within a total analysis time of 10 min. This was achieved even when the highly concentrated dissolution sample was directly injected without the pre-dilution step. Detailed data tables and chromatographic comparisons demonstrating the improved peak symmetry, superior resolution, and shortened analysis time compared with the legacy method are clearly presented in Figure 1 and Table 1.

While modern analytical advancements such as Ultra-High-Performance Liquid Chromatography (UHPLC) or core–shell columns could potentially offer higher theoretical plates and faster separations, conventional HPLC with a standard 5 μm C18 column was deliberately selected for this study. This choice ensures maximum accessibility and seamless method transfer for routine quality control laboratories in generic pharmaceutical industries, where conventional HPLC remains the standard infrastructure. Furthermore, although the mobile phase utilizes standard organic modifiers, the proposed method aligns with sustainable practices by minimizing solvent waste. By reducing the total run time from the 20 min compendial standard to 10 min at a flow rate of 1.2 mL/min, the total solvent consumption and generated chemical waste per injection are reduced by 50%. This reduction provides a more sustainable and economically efficient approach for high-throughput dissolution monitoring without requiring specialized modern instrumentation.

### 2.2. Analytical QbD Approach: ATP, CAA, and PHA

#### 2.2.1. Definition of ATP and CAAs

To systematically develop the analytical method for amiodarone dissolution testing, an AQbD methodology was introduced. First, the ATP elements, which define the primary purpose and required performance characteristics of the method, were established. The fundamental ATP for this study was defined as the development of a robust RP-HPLC method specifically tailored for the quantitative analysis of amiodarone in high-throughput dissolution testing. Based on this defined ATP, the CAAs that directly influence data quality and chromatographic performance were identified according to their risk severity, and specific target criteria were established (Table 2). To ensure accurate quantification without mutual interference, verify column efficiency, and guarantee system consistency, high-critical CAAs were assigned to peak resolution (≥1.5), retention time stability (relative standard deviation (RSD) ≤ 1.0%), tailing factor (≤2.0), symmetry factor (0.8–1.5), and theoretical plate number (≥3000). Furthermore, to secure a robust proportional relationship between sample concentration and detector response—thereby ensuring quantitative reliability across the entire dissolution profile—moderately critical CAAs were defined. These included quantitative response (*R*^2^ ≥ 0.99), accuracy of quantification (recovery of 95.0–105.0%), precision of measurement (peak area RSD ≤ 2.0%), analytical sensitivity, and solution stability (≥72 h stability at room temperature), all of which were successfully integrated into the final development targets.

#### 2.2.2. Preliminary Hazard Analysis

To identify potential risk factors among the chromatographic process variables that could influence the established CAAs, a PHA was conducted as an initial risk assessment. To eliminate subjective bias and ensure robust decision-making, this PHA was not conducted by a single individual; rather, it was executed collaboratively by a cross-functional panel of experts comprising analytical chemists and formulation researchers. When determining the initial risk levels, the panel comprehensively evaluated multiple factors, including prior analytical screening data, the physicochemical properties of amiodarone, the specific matrix effects of the polysorbate 80 surfactant, and established literature on HPLC method development. Variables across the entire analytical method were screened to construct a risk matrix categorized into High, Medium, and Low risk levels based on their severity (Table 3). The risk assessment results reveal that the organic modifier ratio and mobile phase pH were identified as high-risk factors for the majority of chromatographic performance indicators, including resolution, retention time, tailing factor, and symmetry factor. The flow rate presented a high risk regarding retention time and theoretical plate number, while the buffer concentration was determined to be a high-risk factor for the theoretical plate number, which governs column efficiency. Conversely, column temperature was evaluated to exert a medium-risk level of influence across the overall CAA items. Consequently, the PHA matrix screening confirmed that the chemical composition and physical control parameters of the mobile phase could profoundly affect the chromatographic behavior and quantitative reliability of amiodarone. These identified high- and medium-risk variables were subsequently prioritized for a more in-depth, secondary risk assessment utilizing FMEA.

### 2.3. Failure Mode and Effects Analysis

#### 2.3.1. Risk Priority Number Calculation via FMEA

To quantitatively evaluate the severity of the potential risk factors identified in the initial PHA on the CAAs, a FMEA was conducted. Similar to the PHA, this rigorous evaluation was performed collaboratively by the cross-functional expert panel. The panel assigned scores for severity (S), occurrence (O), and detectability (D) on a 1–5 scale based on a consensus derived from empirical observations, historical method validation data, and instrument limitations. Their product was calculated to derive the risk priority number (RPN), where variables with an RPN ≥ 40 were designated as high-risk factors. The FMEA matrix analysis revealed that the organic modifier ratio was evaluated with the highest risk score (RPN = 64); an inappropriate blending ratio of the aqueous and organic phases alters the eluotropic strength, posing a critical risk of baseline overlap between the amiodarone and polysorbate 80 peaks. The mobile phase pH exhibited the next highest risk score (RPN = 60), as inaccurate pH adjustment of the buffer can shift the ionization state of amiodarone, leading to severe peak fronting, peak shape distortion, and failure to meet the symmetry criteria. The flow rate was also flagged as a high-risk factor (RPN = 48) because deviations from the optimal flow rate shift the retention time of the principal analyte and impair chromatographic efficiency and the theoretical plate number. Consequently, these three variables were systematically identified as the high-risk group (Red zone) requiring stringent control (Table 4).

#### 2.3.2. Selection of Critical Parameters for Robustness Testing

Through the FMEA evaluation considering the RPN thresholds, five critical method parameters (CMPs) capable of influencing the CAAs of amiodarone—specifically resolution, retention time, tailing factor, symmetry factor, and theoretical plate number—were ultimately derived. Based on the risk priority number analysis, three variables that exceeded the risk threshold and were classified into the high-risk group were preferentially selected: organic modifier ratio (RPN = 64), mobile phase pH (RPN = 60), and flow rate (RPN = 48). In addition, despite falling below the designated threshold, buffer concentration (RPN = 36) and column temperature (RPN = 24) were included in the final selection, establishing a total of five factors due to their potential to induce variability in residual silanol interactions and stationary phase control based on physicochemical failure modes. These five selected parameters were identified as key factors that govern the established CAAs while remaining susceptible to minor variations in a practical laboratory environment. Consequently, they were definitively adopted as the variable factors for the subsequent robustness testing to statistically validate the reliability and integrity of the proposed analytical method.

### 2.4. Method Validation

#### 2.4.1. System Suitability

As delineated in the ICH Q2 guideline, the system suitability test is a critical component for verifying the performance of an analytical procedure in routine quality control environments. To evaluate the suitability of the finalized and optimized reversed-phase chromatographic system, the amiodarone working standard solution was repeatedly injected six times to confirm the reproducibility of the chromatographic parameters and instrumental stability.

The analytical results demonstrate excellent precision, with the RSD (%) for the amiodarone retention time and peak area calculated as 0.06% and 0.08%, respectively. Furthermore, the mean tailing factor and symmetry factor, which serve as key indicators for evaluating column efficiency and physical peak shapes, were determined to be 0.88 and 1.34, respectively. The mean USP resolution was calculated to be 14.7, measured relative to the immediately preceding peak originating from the polysorbate 80 surfactant matrix. This exceptionally high resolution value confirms the complete baseline isolation of amiodarone from the complex dissolution medium interferences. These values confirmed that the severe peak fronting associated with the legacy USP method (tailing factor of 0.65, symmetry factor of 3.44) was resolved. In addition, the mean theoretical plate number exceeded 5000, representing a substantial improvement in column efficiency compared with the legacy condition (3212) (Table 5). These experimental outcomes met all pre-established system suitability criteria (retention time RSD ≤ 1.0%, peak area RSD ≤ 2.0%, and tailing factor ≤ 2.0). Consequently, these data substantiate that the developed HPLC analytical system is stable and capable of providing highly reliable and consistent data for the practical analysis of dissolution samples.

#### 2.4.2. Specificity

**Figure 2:**
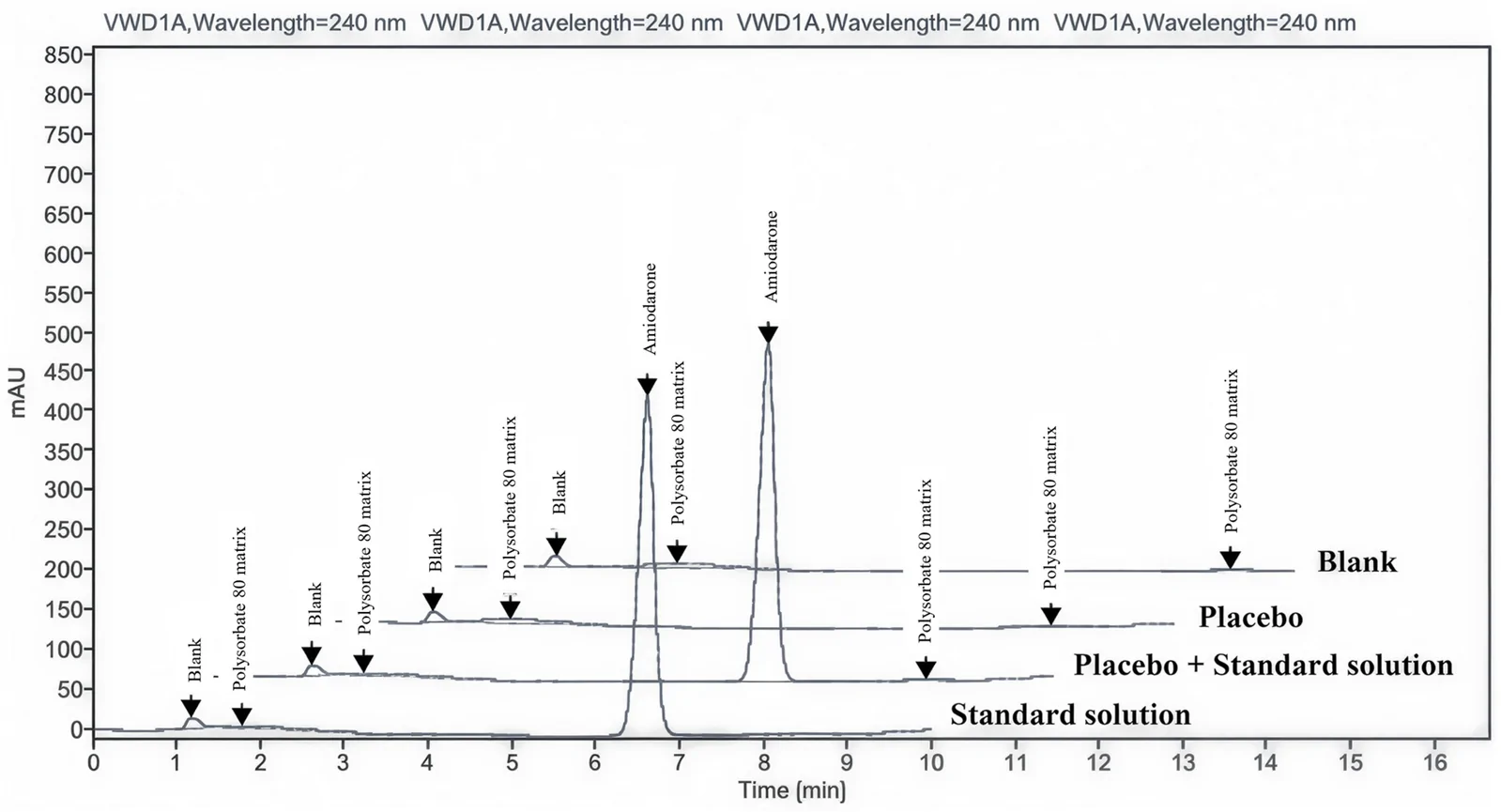
Representative RP-HPLC chromatograms for specificity test.

To verify that the established analytical method can selectively detect amiodarone, the target analyte, within a complex matrix, a specificity evaluation was performed. For this purpose, the blank, placebo, amiodarone working standard, and spiked placebo (Placebo + Standard) solutions were sequentially injected, and their resulting chromatograms were compared and contrasted. The analytical results reveal that in the chromatograms of the blank and placebo solutions, where the formulation excipients and the surfactant (polysorbate 80) were co-present, no absorption peaks or quantitative interferences were observed within the specific retention time window of amiodarone. In contrast, both the working standard and spiked placebo chromatograms exhibited a single, sharp amiodarone peak resolved with high reproducibility at a retention time of approximately 6.6 min (Figure 2). These outcomes demonstrate the exclusion of mutual interferences from the formulation components and the dissolution medium matrix. Consequently, these findings prove that the proposed RP-HPLC method possesses excellent selectivity and analytical validity for the reliable quantification of amiodarone within complex dissolution samples. However, it should be acknowledged as a limitation that the specificity of the current method was evaluated exclusively against formulation excipients and the dissolution medium matrix. Because this method was designed strictly for short-term dissolution monitoring, a comprehensive forced degradation study was not conducted. Given the known susceptibility of amiodarone to photo-degradation and oxidative stress, further verification of specificity against potential degradation products would be required before adapting this analytical method for long-term stability-indicating purposes.

#### 2.4.3. Linearity and Range

**Figure 3:**
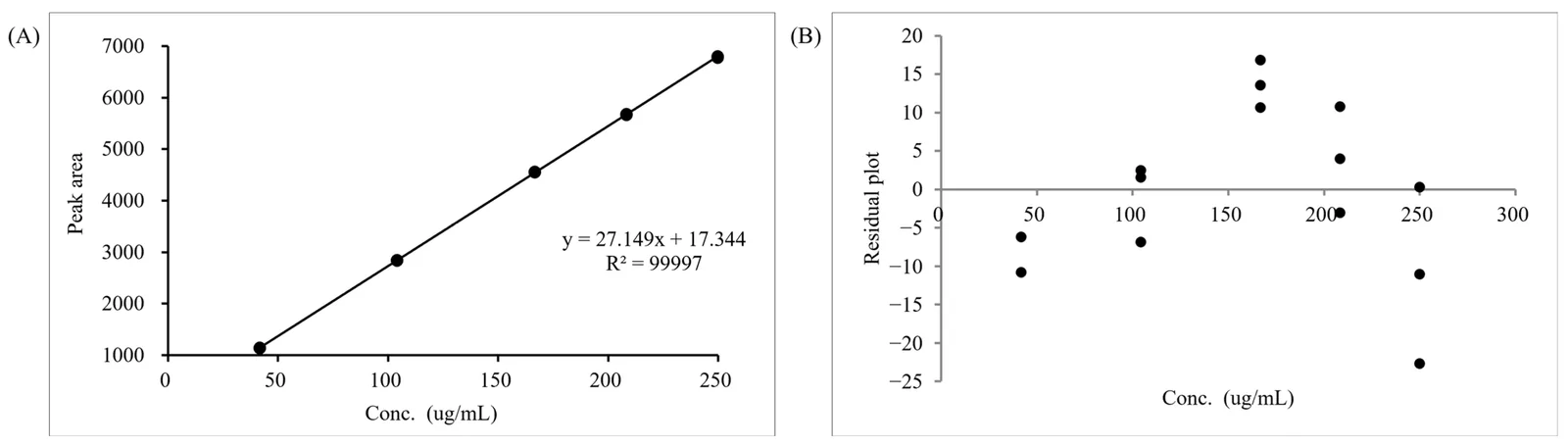
Linearity evaluation of the proposed method: (**A**) Pooled linear calibration curve for amiodarone across the defined working concentration range (*n* = 15). (**B**) Corresponding residual plot demonstrating the random distribution of residuals around zero.

In this study, the linearity of the developed method was evaluated to establish an appropriate range suitable for dissolution profile assessment. To verify linearity, three independent sets of solutions at five concentration levels (44.0, 110.0, 176.0, 220.0, and 264.0 μg/mL), corresponding to 20% to 120% of the target working concentration, were independently prepared from scratch. Instead of evaluating the replicates separately, all data points from these three independent experiments (*n* = 15) were combined into a single pooled linear regression model. The calibration curve was generated by plotting the peak areas against the theoretical concentrations of the working standard solution. Linear regression analysis of the pooled data yielded a coefficient of determination (*R*^2^) of 0.99997, demonstrating excellent linearity across the entire evaluated concentration range (Figure 3 and Table 6). Furthermore, the 95% confidence intervals for the slope and y-intercept were calculated to statistically validate the regression parameters. This outcome satisfied the pre-established acceptance criterion of ≥ 0.99. Furthermore, a residual analysis was conducted to evaluate the appropriateness of the linear model. As illustrated in the residual plot (Figure 3B), the residuals were randomly distributed around the zero axis across all concentration levels without displaying any discernable systematic patterns or trends (e.g., curvature or heteroscedasticity). This random scattering provides robust statistical evidence supporting the true linearity of the method within the specified working range. In addition, according to the ICH guidelines, the range of an analytical method for dissolution testing should encompass ± 20% of the established dissolution specification window. Considering that the routine QC dissolution specification for amiodarone tablets is set at “not less than 80% within 30 min,” the verified linear range of 20% to 120% satisfies and covers these regulatory requirements. In conclusion, these findings substantiate a clear proportional relationship between concentration and peak area within the established range, fulfilling the fundamental prerequisite for the accurate and reliable quantification of the active ingredient in dissolution samples.

#### 2.4.4. Limit of Detection and Limit of Quantitation

The LOD and LOQ for amiodarone were evaluated based on the standard deviation of the response (σ) and the slope of the calibration curve (*S*). The standard deviation of the response was determined using the standard deviation of the y-intercept of the regression line, and the slope was obtained from the linear calibration curve. The LOD, defined as the minimum concentration at which amiodarone can be reliably detected, was calculated to be 0.774 μg/mL according to Equation (1). The LOQ, representing the lowest concentration at which accurate and precise quantification is achievable, was determined to be 2.345 μg/mL based on Equation (2) (Table 7). While these values reflect standard UV detection capabilities, they explicitly demonstrate that the developed analytical method is fit-for-purpose and fully sufficient to monitor and quantify amiodarone even during the early stages of the dissolution testing profile.

#### 2.4.5. Accuracy

To verify the accuracy of the proposed RP-HPLC method, a recovery test was performed to confirm whether amiodarone could be quantified without bias within the dissolution matrix containing the surfactant (polysorbate 80). For the QC accuracy evaluation, spiked samples were prepared by adding the amiodarone working standard to the placebo dissolution matrix at three levels: 80%, 100%, and 120% of the target working concentration, To evaluate the true accuracy of the analytical procedure rather than mere instrument precision, three independent sample solutions were prepared from scratch at each concentration level, resulting in a total of nine independent determinations. The experimental results reveal that the mean recoveries at the 80%, 100%, and 120% levels were 100.11%, 100.03%, and 99.90%, respectively, demonstrating close proximity to the ideal true value (100%) across all concentration zones. The overall mean recovery combining all three concentration levels was 100.01%, and the overall RSD was calculated to be 0.139, confirming extremely low variability in the measured data (Table 8). These quantitative outcomes satisfied the pre-established acceptance criteria range (95.0–105.0% recovery). Consequently, these findings demonstrate that the proposed method is capable of quantifying amiodarone with high accuracy and reliability across the entire analytical range, free from visual or chemical interferences derived from the complex dissolution buffer and formulation excipient matrix.

#### 2.4.6. Precision

To validate the precision of the proposed RP-HPLC method, repeatability and intermediate precision were sequentially evaluated in accordance with the ICH Q2 guideline. First, to assess repeatability, a single analyst independently prepared and analyzed six QC samples corresponding to 100% of the target working concentration. The chromatographic analysis yielded a mean recovery of 99.90% for amiodarone with an RSD of 0.39%. This outcome satisfied the pre-established acceptance criterion (RSD ≤ 2.0%), thereby demonstrating the exceptional instrumental stability and intrinsic precision of the analytical system. For the intermediate precision test to verify the reproducibility of the method across variations in the analytical environment and operators, inter-analyst variability was introduced as the primary environmental factor. A second analyst independently prepared and analyzed six samples on a different day using the identical instrumental system and experimental procedures. The resulting mean recovery was 99.13% with an RSD of 0.47%, maintaining a high level of analytical consistency. Notably, the pooled RSD calculated by combining the datasets from both analysts was determined to be 0.43% (Table 9). This fulfillment of the acceptance criteria (individual and pooled RSD ≤ 2.0%) demonstrates excellent convergence of the data between different analysts. In conclusion, these quantitative results substantiate that the developed RP-HPLC method secures high analytical reliability, enabling the robust and reproducible quantification of amiodarone dissolution samples even under typical laboratory variations.

#### 2.4.7. Robustness

To verify whether the developed analytical method possesses sufficient resistance against minor, unintended experimental variations that may arise in a practical laboratory environment, deliberate variations were introduced into the five critical method parameters selected through the risk assessment. The variable factors included the mobile phase organic modifier ratio, pH, flow rate, column temperature, and TEA concentration in the buffer. The specific variation ranges for each condition and the corresponding quantitative results of the chromatographic performance indicators—comprising retention time, recovery, SD, RSD, percentage difference, tailing factor, symmetry factor, resolution, and theoretical plate number—are described in detail in Table 10, Table 11, Table 12, Table 13 and Table 14, respectively. As a result of the comprehensive quantitative evaluation, the mean recovery of amiodarone remained stably within the range of 98.39–101.26% under all stress conditions. Furthermore, both the intra-day precision (RSD ≤ 1.28%) and the percentage difference relative to the standard conditions (Difference ≤ 1.69%) clearly satisfied the pre-established acceptance criteria (≤2.0% and ≤3.0%, respectively), thereby demonstrating the intrinsic quantitative reliability of the method.

However, in terms of chromatographic separation behavior, variations in the buffer pH (yielding symmetry factors of 0.69 and 2.14 at pH 3.8 and 4.2, respectively) and an increase in column temperature (resulting in a decrease in resolution to 2.1 at 55 °C) were identified as critical method parameters that exert a relatively sensitive influence on peak symmetry and resolution efficiency. Notably, the symmetry factors observed at pH 3.8 and 4.2 strictly fall outside our pre-established acceptance criteria (0.8–1.5), representing a failure in system suitability for peak symmetry under these extreme conditions. This sensitivity is attributed to the ionization characteristics of amiodarone and its thermodynamic interactions with the stationary phase reacting highly sensitively to changes in pH. Therefore, we openly acknowledge that this method possesses a narrow pH tolerance window, which serves as a genuine robustness limitation of the current procedure. To prevent peak distortion and maximize data reproducibility during future large-scale routine analyses and method transfers, strict and precise control over mobile phase pH adjustment must be mandated in the operational protocol. Conversely, under minor fluctuations in the mobile phase flow rate, organic modifier ratio, and TEA concentration, no deterioration in peak shape or separation efficiency was observed, apart from predictable physical shifts in retention time. However, it should be acknowledged as a methodological limitation that the robustness evaluation for the mobile phase composition (Table 10) involved simultaneous variations in acetonitrile, methanol, and the buffer solution to maintain the 100% total volumetric ratio. Consequently, the variations observed in separation efficiency, such as the decreased resolution to 8.1 in Condition 3, represent a combined effect of altered organic modifier strength and reduced buffer proportion rather than a purely univariate response. Nevertheless, despite this multivariate stress, all system suitability parameters under these conditions remained perfectly within their acceptance thresholds (resolution ≥ 1.5), demonstrating adequate buffering capacity. In conclusion, it was confirmed that the proposed analytical method maintains robustness and analytical integrity against routine laboratory variability, provided that appropriate control strategies for the sensitive parameters are incorporated into the operational protocol.

#### 2.4.8. Stability in Dissolution Medium

The evaluation of solution stability was performed to assess the chemical stability of amiodarone during the dissolution testing process and subsequent sample storage under typical laboratory conditions. The working standard and dissolution sample solutions were prepared and stored at room temperature (25 ± 2 °C, 60% ± 5% relative humidity (RH)), and chromatographic analyses were executed at pre-established time points: initial, 12, 24, 48, and 72 h. The stability of the solutions was evaluated by calculating the percent recovery (%) and percentage difference (%) at each time interval relative to the initial analytical results, while the precision of the measured data was verified via the RSD (%). The acceptance criteria for solution stability were defined as a mean recovery within 95.0–105.0%, an RSD of ≤2.0% for each individual time point, and a percentage difference of ≤3.0% compared with the initial value. The experimental results confirm that both the amiodarone working standard and dissolution sample solutions remained stable for up to 72 h under ambient room temperature conditions. Throughout the 72 h run, the chromatographic repeatability (RSD) of the dissolution sample solution at each time interval ranged from 0.03% to 0.23%, satisfying the system suitability thresholds. Furthermore, at the final 72 h time point, the recovery of the dissolution sample solution was determined to be 101.56%, representing a percentage difference of merely 1.87% relative to the initial value, which complied with the pre-established robustness criteria (Table 15). These findings demonstrate that the active pharmaceutical ingredient, amiodarone, retains its chemical integrity without degradation or adsorption issues, even upon prolonged exposure within the complex dissolution medium containing the surfactant. In conclusion, the developed RP-HPLC method is proven to be highly suitable for reliable high-throughput quantitative analysis, effectively eliminating concerns regarding solution instability-induced errors during the sample dwell times or autosampler standby periods typical of large-scale dissolution studies.

### 2.5. Application to Dissolution Studies

**Figure 4:**
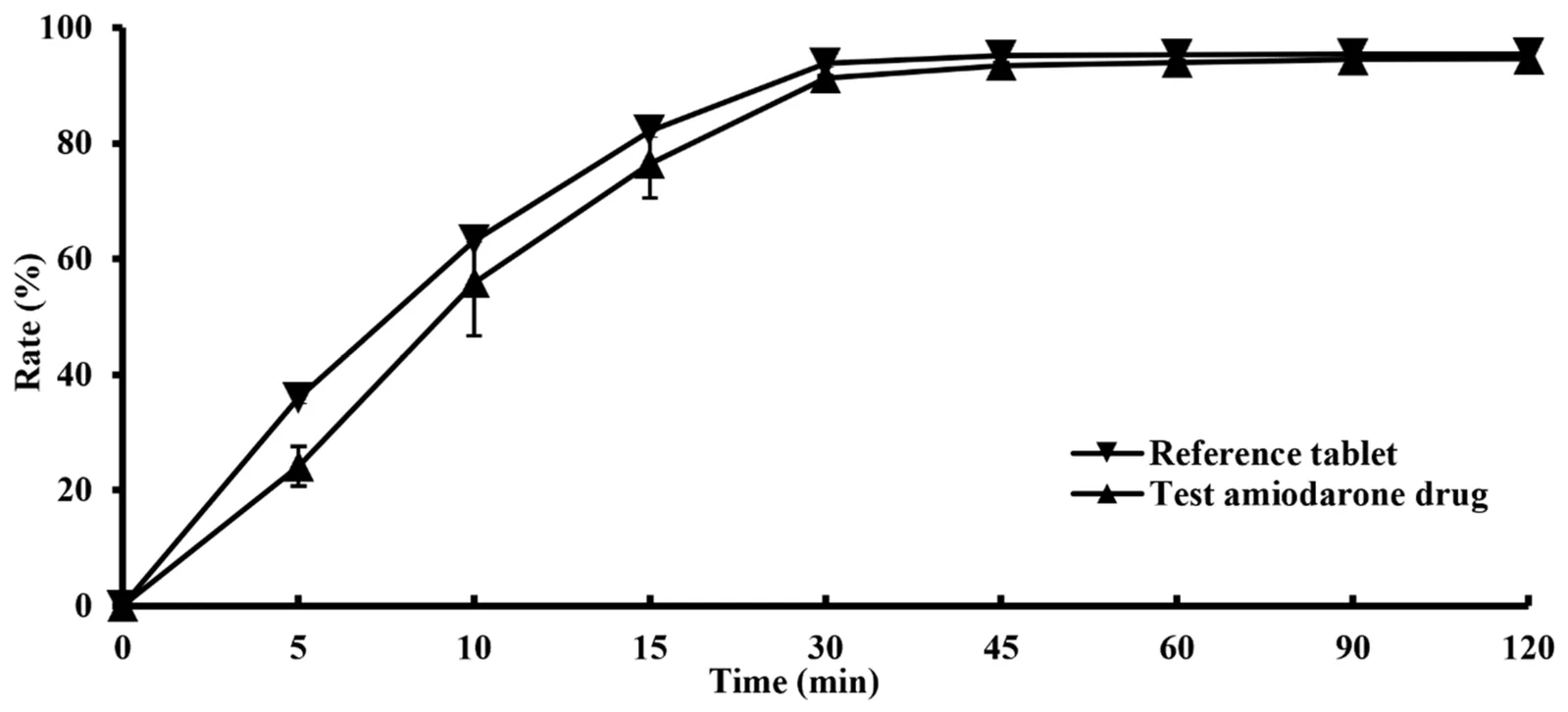
Dissolution profiles of the test amiodarone tablets and the reference product (Cordarone tablets).

The RP-HPLC method developed and validated in this study was applied to the actual dissolution testing of amiodarone tablets to verify its field applicability for high-throughput quantitative dissolution sample analysis. The dissolution test was performed under pre-established standard operational conditions, and the dissolution samples collected at each designated time point were analyzed using the proposed HPLC method. The evaluation of the dissolution profiles revealed that both the reference and newly developed test formulations exhibited excellent dissolution characteristics typical of immediate-release dosage forms. Both formulations demonstrated a steep upward dissolution curve up to the initial 15 min and achieved a dissolution rate of over 80% at the 30 min time point, which serves as the critical quality control milestone in this study (Figure 4). Despite the presence of potentially interfering substances derived from the complex surfactant matrix and formulation excipients, the analytical method was capable of quantifying amiodarone stably and reliably without significant errors throughout the entire 120 min dissolution process. In conclusion, these outcomes demonstrate that the developed RP-HPLC method can track the intrinsic dissolution characteristics of the formulation, proving its suitability for application in the large-scale routine QC and product release testing of amiodarone formulations.

Beyond the specific application to amiodarone, the analytical strategies and AQbD learning established in this study offer broader implications for pharmaceutical analysis. Many highly lipophilic active pharmaceutical ingredients (BCS Class II and IV) necessitate the use of high-concentration surfactant media to maintain adequate sink conditions during dissolution testing. This universally introduces severe chromatographic matrix interferences and often forces analysts to adopt inefficient pre-dilution steps. The systematic framework demonstrated here—utilizing PHA and FMEA to proactively identify the risk of surfactant-analyte peak overlap, followed by targeted mitigation through isocratic eluotropic strength and pH adjustments—provides a highly transferable blueprint. By proving that massive surfactant interference can be completely bypassed chromatographically without sample dilution, this AQbD-driven approach can be readily adapted to streamline dissolution method development for a wide array of complex, surfactant-heavy pharmaceutical formulations, ultimately enhancing high-throughput screening capabilities across the generic pharmaceutical industry.

## 3. Materials and Method

### 3.1. Chemicals and Reagents

Amiodarone hydrochloride, used as the active pharmaceutical ingredient (API) in this study, was obtained from Zhejiang Hengkang Pharmaceutical Co., Ltd. (Taizhou, Zhejiang, China). Among the primary excipients utilized for the formulation design, lactose monohydrate 200 M (Pharmatose 200 M) and pregelatinized starch 1500 were supplied by DFE Pharma (Goch, North Rhine-Westphalia, Germany) and Colorcon Korea (Suwon, Gyeonggi-do, Republic of Korea), respectively. Additionally, povidone K25 (BASF, Ludwigshafen, Rhineland-Palatinate, Germany), colloidal silicon dioxide (Aerosil 200, Evonik, North Rhine-Westphalia, Germany), and magnesium stearate (Nitika Pharmaceutical Specialties Pvt. Ltd., Maharashtra, Maharashtra, India) were procured as binders and additive components for the experimental procedures. Acetonitrile and methanol, used as the organic solvents for HPLC analysis, were purchased from Duksan Pure Chemicals (Ansan, Gyeonggi-do, Republic of Korea) in HPLC grade. All other chemicals and reagents were of analytical grade and used as received from commercial sources without further purification.

### 3.2. ATP and Risk Assessment Approach

To monitor the dissolution samples of amiodarone tablets, an ATP was established to define the desired performance characteristics of the proposed RP-HPLC method. Based on the ATP, CAAs were identified to guarantee the accuracy, precision, specificity, and resolution of the method [27,28]. Subsequently, a risk assessment utilizing PHA and FMEA was performed to evaluate the impact of potential variable factors arising during mobile phase preparation and instrument operation—such as mobile phase composition, pH, flow rate, column temperature, and TEA concentration—on the performance of the method [29,30]. Through this systematic approach, CMPs that could induce significant deviations in peak symmetry and separation efficiency were identified, and variable stress conditions were established for the subsequent robustness testing.

### 3.3. Instrumentation and Chromatographic Conditions

Quantitative analysis of amiodarone was performed using an HPLC system equipped with an ultraviolet–visible (UV-Vis) detector (Agilent 1260 Infinity II, Agilent Technologies, Santa Clara, CA, USA). Chromatographic separation was achieved on a Zorbax Eclipse XDB-C18 column (Agilent Technologies, Santa Clara, CA, USA) (4.6 × 150 mm, 5 μm), with the detection wavelength set at 240 nm. The mobile phase was prepared by mixing acetonitrile, methanol, and buffer in a ratio of 45:27:28 (*v*/*v*/*v*). The buffer solution was prepared by adding 5 mL of TEA to 1 L of distilled water, followed by precise pH adjustment to 4.0 using phosphoric acid. To maximize analytical efficiency, the flow rate was maintained at 1.2 mL/min, the column oven temperature was kept at 50 °C, and the sample injection volume was set at 10 μL.

### 3.4. Preparation of Dissolution Medium

The dissolution medium was prepared by adding polysorbate 80, a non-ionic surfactant, to an acetate buffer. First, 3.0 g of sodium acetate and 6 mL of glacial acetic acid were introduced into a 1 L beaker and dissolved in a small volume of purified water, after which additional purified water was added to bring the final volume to 1 L. The final pH of the prepared buffer was adjusted to 4.0 using glacial acetic acid, if necessary. Subsequently, an approximately 20% aliquot (200 mL) of the prepared buffer was taken, and 10 g of polysorbate 80 was added. The mixture was then homogeneously dissolved using an ultrasonicator (SD-301H, MUJIGAE, Seoul, Republic of Korea) to ensure complete micelle formation. Once the solution became completely clear, it was blended with the remaining 800 mL of the buffer and stirred thoroughly to be utilized as the final dissolution medium.

### 3.5. Preparation of Analytical Solutions

#### 3.5.1. Standard and QC Solutions

The stock standard solution for the quantitative analysis and calibration curve construction of amiodarone was prepared by weighing 220 mg of amiodarone hydrochloride and transferring it into a 100 mL volumetric flask. Approximately 50 mL of the dissolution medium was added to the flask, and sonication was performed until the drug was completely dissolved. After the solution cooled to room temperature, it was diluted to volume with the same dissolution medium to yield a stock standard solution with a concentration of 2.2 mg/mL. The working standard solution for analysis was prepared by diluting the aforementioned stock standard solution with the dissolution medium to achieve a final concentration of 0.22 mg/mL. To minimize interference during instrumental analysis, the solution was filtered through a 0.45 μm regenerated cellulose (RC) syringe filter (Sartorius, Göttingen, Lower Saxony, Germany). To control potential compositional errors caused by drug adsorption onto the filter membrane, the initial filtrate was discarded, and only the subsequent filtrate was collected and used as the working standard and QC samples for the experiments.

#### 3.5.2. Placebo and Matrix Solutions

To evaluate the specificity of the analytical method, a placebo stock solution was prepared using an excipient mixture excluding amiodarone, the API. A total of 250 mg of the excipient mixture, corresponding to the formulation of a single tablet (lactose monohydrate, pregelatinized starch, povidone K25, magnesium stearate, and colloidal silicon dioxide) was weighed and transferred into a 100 mL volumetric flask. These selected excipients are qualitatively identical to the inactive ingredients comprising the reference innovator product (Cordarone tablets). Because the excipient profile is common to both the investigational formulation and the reference product, no further excipients were required to be evaluated for matrix interference. After adding 50 mL of the dissolution medium, sonication was performed to ensure sufficient dispersion and extraction of the excipient components. Once the mixture reached room temperature, it was diluted to volume with the same dissolution medium to adjust the final volume to 100 mL.

#### 3.5.3. Dissolution Samples

The dissolution test was performed in accordance with USP Dissolution Test Method II (Paddle method) (708-DS Dissolution Apparatus, Agilent, Santa Clara, CA, USA). A single amiodarone tablet was introduced into 900 mL of the pH 4.0 dissolution medium maintained at 37 ± 0.5 °C, followed by agitation at 75 rpm. At a time point of 30 min after the initiation of the test, a 4 mL aliquot of the dissolution medium was withdrawn. The collected sample was filtered through a 0.45 μm RC syringe filter to remove insoluble excipients. At this stage, consistent with the aforementioned preparation of the working standard solution, the initial portion of the filtrate was discarded to eliminate potential adsorption interference of the active ingredient onto the filter membrane. The subsequent filtrate was then utilized as the sample solution for the HPLC analysis.

### 3.6. Method Validation

To establish the validity and objective reliability of the newly developed RP-HPLC dissolution method for immediate-release amiodarone tablets, validation was conducted in accordance with the ICH Q2(R1) guidelines. This verification procedure focused on confirming that the optimized analytical conditions were suitable for their intended purpose and capable of yielding consistent data without errors under repetitive operational environments. As key validation parameters, system suitability, specificity, linearity, analytical range, accuracy, precision, LOD, LOQ, robustness, and solution stability were comprehensively evaluated. Notably, to ensure the practical feasibility and field applicability of the proposed method, both pure standard solutions and samples containing the actual dissolution matrix were evaluated in parallel, thereby cross-validating the universal applicability of the analytical method.

#### 3.6.1. System Suitability

Because variable factors that may arise within a chromatographic system can compromise the reliability of analytical data, a system suitability test was conducted to pre-verify system performance. This test aimed to objectively confirm the sensitivity, resolution, and reproducibility of the analytical system. To evaluate system suitability, the amiodarone working standard solution was employed as the analytical sample, and data were acquired through six replicate injections. The acceptance criteria were established as an RSD of ≤2.0% for the amiodarone peak area, a tailing factor of ≤2.0, and an RSD of ≤1.0% for the retention time of the amiodarone peak to comprehensively assess the suitability of the analytical system.

#### 3.6.2. Specificity

To verify that the proposed method can independently identify the amiodarone peak without interference from other components in the sample matrix, chromatograms of the blank, placebo, working standard, and sample solutions were compared and analyzed. For the evaluation of specificity, the placebo solution was prepared by transferring 11 mL of the placebo stock solution into a 100 mL volumetric flask, followed by diluting to volume with the dissolution medium. The sample solution was prepared in the same manner as the QC sample preparation described in Section 3.5.1. Prior to instrumental analysis, all test solutions were filtered through a 0.45 μm RC syringe filter to remove microparticles and insoluble excipients from the samples. At this stage, to prevent any quantitative loss of the active ingredient due to the filter membrane, the initial portion of the filtrate was discarded, and only the subsequently collected filtrate was subjected to analysis to evaluate the presence of interferences at the retention time of the amiodarone peak.

#### 3.6.3. Linearity and Range

To establish the linearity and analytical range of the method, the ICH guideline recommendation of “±20% of the established dissolution specification range in the quality standard of the formulation” was applied. Considering the QC dissolution specification of the formulation, which is “not less than 80% dissolution within 30 min,” the range was set to encompass 20% to 120% of the final target working concentration. Accordingly, five concentration levels of amiodarone (44.0, 110.0, 176.0, 220.0, and 264.0 μg/mL) were selected. To ensure the utmost reliability and evaluate true method reproducibility rather than mere instrument precision, three independent calibration curves were constructed. For each curve, a fresh set of calibration standard solutions was independently prepared from scratch, resulting in three independent experiments (Table 16). A calibration curve was constructed by plotting the analyte concentration on the x-axis against the corresponding peak area on the y-axis, and linear regression analysis was performed using the least-squares method. The suitability of the method was evaluated based on a coefficient of determination (*R*^2^) criterion of ≥0.99 to demonstrate the statistical correlation between the concentration and the analytical response.

#### 3.6.4. Limit of Detection and Limit of Quantitation

To evaluate the sensitivity of the analytical method, the LOD and LOQ were determined. In this study, the theoretical calculation approach recommended by the ICH Q2 guideline was applied, which is based on the statistical relationship between the slope of the calibration curve and the standard deviation of the response. The standard deviation of the response (*σ*) was estimated from the standard deviation of the y-intercept of the linear regression equation. The final threshold values were derived by multiplying the ratio of the standard deviation to the slope of the calibration curve (*S*) by factors of 3.3 for LOD and 10 for LOQ, using the following equations:

LOD = 3.3 × *δ*/*S* (1)

LOQ = 10 × *δ*/*S* (2)

#### 3.6.5. Accuracy

The accuracy of the analytical method was verified by determining the recovery percentage of spiked samples, prepared by adding known amounts of the amiodarone hydrochloride active ingredient to a placebo matrix. The test samples were prepared at three concentration levels corresponding to 80%, 100%, and 120% of the target concentration (176.0, 220.0, and 264.0 μg/mL). At each concentration level, three independent samples were prepared by separately spiking the placebo matrix, yielding nine independent determinations in total (Table 17). Accuracy was evaluated based on the mean recovery percentage of amiodarone derived from the prepared samples, and the acceptance criterion was established within 100 ± 5% to ensure the trueness of the analytical data.

#### 3.6.6. Precision

The precision of the analytical method was evaluated to assess the consistency and reproducibility of the measured values under both identical conditions and varied analytical environments. In this study, precision was verified by subdividing it into repeatability and intermediate precision. To evaluate repeatability, six independently prepared samples at the target working concentration (220 μg/mL) were analyzed. Furthermore, intermediate precision was assessed to confirm the impact of typical variations that may occur within the laboratory. The intermediate precision test was performed using the same RP-HPLC system but involved independent sample preparations and analyses conducted by different analysts on different days using identical procedures. The suitability of the method was evaluated by calculating the RSD of the amiodarone peak areas obtained under each testing condition. The acceptance criteria were established as ≤2.0% for both the individual RSDs under each analytical condition and the pooled RSD combining the datasets from both analysts, thereby demonstrating the robustness and quantitative reliability of the method against intralaboratory variations.

#### 3.6.7. Robustness

To verify whether the analytical method possesses sufficient resistance against minor experimental variations that may occur in a practical laboratory environment, a robustness test was performed. The deliberate variations and their respective ranges were established as follows: organic modifier ratio (±2.0%), mobile phase pH (±0.2), flow rate (±0.1 mL/min), column temperature (±5 °C), and TEA concentration in the buffer (0.5% ± 0.05%, *v*/*v*). Under each altered condition, a single concentration of the amiodarone working standard and sample solutions was repeatedly injected to monitor the retention time and quantitative recovery results, which were subsequently evaluated by comparing them with the outcomes obtained under the initially optimized standard conditions. The acceptance criterion for system suitability was defined as an RSD of ≤2.0% for the amiodarone peak area calculated within each varied condition. Furthermore, the percentage difference (%) in the measured values under the varied conditions relative to the standard conditions was calculated using Equation (3). The acceptance threshold for this difference was set at ≤3.0% to confirm that the proposed analytical method maintains excellent reliability and robustness without significant fluctuations despite minor changes in the analytical environment.

(3) $$Difference\;\left(\%\right)=\left|\frac{{Value}_{altered}-{Value}_{optimized}}{{Value}_{optimized}}\right|\times 100$$

where Value_optimized_ represents the measured value under the initially optimized standard conditions, and Value_altered_ denotes the measured value under the deliberately varied conditions.

#### 3.6.8. Stability in Dissolution Medium

The evaluation of solution stability in the dissolution medium was performed to ensure the chemical stability of the amiodarone working standard and sample solutions under controlled conditions over time. This test was conducted using storage time as a variable factor, with each solution stored under ambient room temperature conditions (25 ± 2 °C, 60 ± 5% RH). The concentration of amiodarone in both the standard and sample solutions (prepared at the 100% target working concentration of 220.0 μg/mL) was monitored immediately after preparation (initial) and at designated intervals of 12, 24, 48, and 72 h using the developed RP-HPLC method. The stability of the solutions was evaluated by comparing the percentage difference (%) between the measured value at each time point and the initial value, which was calculated according to Equation (3) described in the robustness section. The acceptance criteria for solution stability were established as a difference of ≤3.0% relative to the initial measured value and an RSD of ≤2.0% for the replicate injections at each time point, thereby verifying the stability of the solutions over time.

## 4. Conclusions

In this study, a novel RP-HPLC method featuring enhanced analytical performance compared with the legacy USP method was successfully developed and validated for the efficient and reliable quality control of immediate-release amiodarone formulations. The newly developed method not only shortens the long retention time of the conventional compendial method (from over 20 min to approximately 10 min) but also secures optimal sensitivity, offering the advantage of direct sample injection without requiring any pre-dilution steps. This advancement streamlines sample preparation and analytical cycle times during large-scale routine analyses, eliminates potential dilution errors at the source, and ensures high operational efficiency. Through a comprehensive method validation program conducted in strict accordance with the ICH Q2 guidelines, the quantitative reliability and overall system validity of the proposed method were established across all evaluated parameters (specificity, linearity, precision, and solution stability). Notably, the results from the repeatability tests, inter-analyst intermediate precision trials, and the 72 h long-term solution stability assessment within the dissolution medium support the capacity of the analytical system to maintain excellent consistency and chemical integrity regardless of environmental fluctuations. Furthermore, a multifaceted robustness evaluation based on a systematic risk assessment comprehensively verified that the method possesses sufficient resistance and integrity against minor experimental stress variations in the mobile phase composition, pH, flow rate, column temperature, and TEA concentration. However, the evaluation also clearly demonstrated that the method is not robust against variations in mobile phase pH and column temperature. Specifically, minor fluctuations in pH caused the peak symmetry to fall outside the acceptable system suitability criteria, indicating a narrow pH tolerance window. We explicitly acknowledge this sensitivity to pH and temperature as a genuine limitation of the proposed method. Consequently, integrating a precise and strict control strategy for these two critical variables into the operational protocol is absolutely mandatory to prevent peak distortion and ensure data validity. Finally, the developed method was successfully applied to actual in vitro dissolution testing of the reference (Cordarone tablet) and test formulations, precisely tracking the unique dissolution kinetics and regulatory compliance of the immediate-release dosage forms without any interference from the complex surfactant matrix. In conclusion, the proposed RP-HPLC method combines outstanding analytical reliability, excellent field applicability, and economic efficiency, rendering it a practical tool for the high-throughput quality control and formulation optimization studies of amiodarone products.

## Figures and Tables

**Table 1:** Comparison of analytical parameters and chromatographic performance between the legacy USP method and the proposed RP-HPLC method.

Analytical Parameter	Legacy USP Method	Proposed RP-HPLC Method	Improvement/Advantage
Sample Preparation	10-fold dilution required	Direct injection	Eliminates pre-treatment step, reduces potential dilution errors
Run Time	20 min	10 min	~50% reduction, enables high-throughput routine QC
Tailing Factor	0.65	0.88	Resolves severe peak fronting
Symmetry Factor	3.44	1.33	Ensures normal, symmetrical peak elution
Resolution (from matrix)	1.80	14.6	Complete baseline isolation from polysorbate 80
Theoretical Plates	3212	5294	Improved column efficiency

Note: The chromatographic values for the proposed method represent the average outcomes established during the system suitability evaluation.

**Table 2:** Analytical target profile elements and identified CAAs for amiodarone dissolution method.

ATP Elements	Target	Is This a CAA?	Justification
Resolution	Baseline resolution (≥1.5) from adjacent impurity/degradation peaks.	Yes	Ensures accurate quantification of amiodarone without mutual interference.
Retention time	Consistent retention time (RSD ≤ 1.0%) for proper peak identification.	Yes	Demonstrates system consistency and ensures accurate identification of the analyte.
Tailing factor	Tailing factor for the amiodarone peak ≤ 2.0.	Yes	Limits peak asymmetry to reduce integration errors and ensure accuracy.
Symmetry factor	Peak symmetry factor maintained within 0.8–1.5.	Yes	Verifies normal column condition and symmetrical peak elution.
Theoretical plate number	Theoretical plate number for the amiodarone peak ≥ 3000.	Yes	Verifies sufficient column efficiency for routine analytical operation.
Quantitative response	Linear response with *R*^2^ ≥ 0.99 across the dissolution testing range.	Yes	Ensures a proportional relationship between concentration and detector response.
Accuracy of quantification	Recovery of 95.0–105.0% relative to the nominal concentration.	Yes	Ensures closeness between measured and true values within dissolution limits.
Precision of measurement	Peak area RSD ≤ 2.0% for repeatability and intermediate precision.	Yes	Demonstrates reproducibility during serial analysis of sample batches.
Analytical sensitivity	LOD and LOQ sufficiently lower than the earliest dissolution timepoints.	Yes	Ensures reliable quantification across the entire dissolution profile.
Sample solution stability	Standard and sample solutions stable for ≥72 h at room temperature.	Yes	Ensures analyte integrity during sequence runs and autosampler wait times.

Note: High-critical attributes are highlighted in red, and moderately critical attributes in orange.

**Table 3:** Preliminary hazard analysis matrix for amiodarone dissolution method.

CAAs	Organic Modifier Ratio	Mobile Phase pH	Flow Rate	Column Temperature	Buffer Concentration
Resolution	High	High	Medium	Medium	Medium
Retention time	High	High	High	Medium	Low
Tailing factor	High	High	Low	Medium	Medium
Symmetry factor	High	High	Low	Medium	Medium
Theoretical plate number	Medium	Medium	High	Medium	High
Quantitative response	High	Medium	Low	Low	Low
Accuracy of quantification	Medium	Medium	Low	Low	Medium
Precision of measurement	Medium	Medium	Medium	Medium	Medium
Analytical sensitivity	Medium	Low	Low	Low	Low
Sample solution stability	Low	Low	Low	Low	Low

*Note: High-critical attributes are highlighted in red, moderately critical attributes in orange, and non-critical attributes in green.*

**Table 4:** Failure mode and effects analysis for amiodarone dissolution method.

Process Variables	Failure Mode	Potential Failure Effects on CAAs	S	O	D	RPN
Organic modifier ratio	Incorrect ratio of aqueous and organic components	Changes in organic modifier ratio directly shift elution strength, potentially leading to baseline overlap between amiodarone and Polysorbate 80 peaks.	4	4	4	64
Mobile phase pH	Inaccurate pH adjustment of buffer solution	Incorrect pH alters the ionization state of amiodarone, leading to critical peak distortion, severe fronting, and failure in symmetry criteria.	5	4	3	60
Flow rate	Deviation from optimal flow rate	Flow rate variations shift the retention time of the target analyte and compromise chromatographic efficiency and theoretical plate numbers.	4	3	4	48
Buffer concentration	Inappropriate buffer salt concentration	Incorrect ionic strength fails to mask residual silanol interactions, potentially degrading peak resolution and theoretical plates.	3	3	4	36
Column temperature	Temperature fluctuation	Column temperature changes slightly affect analyte–stationary phase interactions, leading to minor shifts in peak shape and retention consistency.	3	2	4	24

Abbreviations: S, severity; O, probability of occurrence; D, detectability; RPN, risk priority number. Each parameter was scored on a 1–5 scale, and the RPN was calculated as S × O × D. Parameters with an RPN value ≥ 40 were considered high-risk factors and highlighted in red.

**Table 5:** Chromatographic parameters and system suitability outcomes for amiodarone standard solution injections.

Standard Solution Number	Retention Time (min)	Area	Tailing Factor	Symmetry Factor	Resolution	Theoretical Plate Number
1	6.61	5670.289	0.88	1.34	14.3	5224
2	6.60	5678.650	0.89	1.33	14.6	5094
3	6.61	5677.002	0.88	1.33	14.8	5247
4	6.61	5673.994	0.89	1.34	14.6	5195
5	6.61	5671.526	0.88	1.34	15.0	5186
6	6.61	5665.621	0.88	1.34	15.2	5193
Average (%)	6.61	5672.847	0.88	1.34	14.7	5190
Standard deviation (SD)	0.00	4.75	0.00	0.00	0.33	52.21
RSD (%)	0.06	0.08	0.31	0.25	2.22	1.01

Note: The USP resolution values were calculated relative to the immediately preceding interference peak derived from the polysorbate 80 dissolution matrix.

**Table 6:** Linear regression parameters and data for the amiodarone dissolution analytical method.

Concentration (%)	Peak Area				95% CI
	Test 1	Test 2	Test 3	Average	
20	1139.799	1144.176	1139.600	1141.192	-
50	2847.986	2847.101	2838.648	2844.578	
80	4551.274	4557.500	4554.231	4554.335	
100	5674.697	5681.488	5667.658	5674.614	
120	6778.110	6789.766	6801.116	6789.664	
Slope	27.107	27.148	27.193	27.149	27.039 to 27.229
Y-intercept	21.331	20.700	10.002	17.344	3.471 to 37.074
*R* ^2^	0.99997	0.99997	0.99999	0.99997	-

**Table 7:** Limit of detection and limit of quantitation for the amiodarone analytical method.

API	σ	*S*	LOD (μg/mL)	LOQ (μg/mL)
Amiodarone Hydrochloride	6.366	27.149	0.774	2.345

**Table 8:** Accuracy and recovery results for the amiodarone analytical method.

Level (%)	Corrected Concentration (μg/mL)	Found Concentration (μg/mL)	Accuracy (%)	Average (%)
80	166.59	166.66	100.04	100.11
		166.69	100.06	
		166.96	100.22	
100	208.23	207.90	99.84	100.03
		208.37	100.07	
		208.61	100.18	
120	249.88	249.64	99.90	99.90
		249.50	99.85	
		249.73	99.94	
Grand average (%)				100.01
Grand RSD (%)				0.139

Found concentration (µg/mL) = (QC sample peak area − intercept)/slope. Accuracy = Found concentration/Corrected concentration × 100.

**Table 9:** Repeatability and intermediate precision results for the amiodarone analytical method.

Number of Determinations (*n*)	Repeatability	Intermediate Precision
1	99.96	99.32
2	100.23	99.28
3	100.24	99.62
4	100.12	98.66
5	99.42	98.45
6	99.40	99.42
Average (%)	99.90	99.13
RSD (%)	0.39	0.47
Inter-analyst RSD (%)	0.43	

**Table 10:** Robustness testing results for amiodarone under mobile phase composition variations.

Parameter		Standard (45:27:28)	Condition 1 (47:25:28)	Condition 2 (43:29:28)	Condition 3 (46:28:26)	Condition 4 (44:26:30)
Recovery (%)	1	99.99	101.68	99.46	100.48	99.22
	2	100.14	100.96	97.84	100.22	99.40
	3	100.11	101.13	97.86	100.17	99.82
Average (%)		100.08	101.26	98.39	100.29	99.48
Standard deviation (SD)		0.08	0.37	0.93	0.17	0.31
RSD (%)		0.08	0.37	0.94	0.17	0.31
Difference (%)		-	1.18	1.69	0.21	0.60
Retention time (min)		6.61	6.62	7.04	6.17	7.44
Tailing factor		0.89	1.07	1.02	0.88	1.18
Symmetry factor		1.34	0.94	1.01	0.88	0.80
Resolution		14.6	13.6	13.4	8.1	14.0
Theoretical plate number		5189	5357	5044	4269	4872

Note: The ratios in parentheses represent the volumetric composition of acetonitrile, methanol, and buffer (*v*/*v*/*v*), respectively.

**Table 11:** Robustness testing results for amiodarone under mobile phase pH variations.

Parameter		Standard (pH 4.0)	pH 3.8	pH 4.2
Recovery (%)	1	99.99	99.98	100.28
	2	100.14	99.77	100.13
	3	100.11	99.64	100.23
Average (%)		100.08	99.80	100.21
Standard deviation (SD)		0.08	0.17	0.08
RSD (%)		0.08	0.17	0.07
Difference (%)		-	0.28	0.13
Retention time (min)		6.61	5.83	8.49
Tailing factor		0.89	1.31	0.75
Symmetry factor		1.34	0.69	2.14
Resolution		14.6	9.0	7.6
Theoretical plate number		5189	4214	3742

**Table 12:** Robustness testing results for amiodarone under flow rate variations.

Parameter		Standard (1.2 mL/min)	1.1 mL/min	1.3 mL/min
Recovery (%)	1	99.99	99.33	98.79
	2	100.14	99.65	98.80
	3	100.11	99.60	99.03
Average (%)		100.08	99.53	98.87
Standard deviation (SD)		0.08	0.18	0.14
RSD (%)		0.08	0.18	0.14
Difference (%)		-	0.55	1.21
Retention time (min)		6.61	7.37	6.21
Tailing factor		0.89	1.01	1.08
Symmetry factor		1.34	1.03	0.93
Resolution		14.6	13.8	7.2
Theoretical plate number		5189	5349	4552

**Table 13:** Robustness testing results for amiodarone under column temperature variations.

Parameter		Standard (50 °C)	45 °C	55 °C
Recovery (%)	1	99.99	100.23	98.23
	2	100.14	100.22	100.22
	3	100.11	100.42	100.60
Average (%)		100.08	100.29	99.68
Standard deviation (SD)		0.08	0.12	1.28
RSD (%)		0.08	0.12	1.28
Difference (%)		-	0.21	0.84
Retention time (min)		6.61	6.95	6.73
Tailing factor		0.89	1.30	0.94
Symmetry factor		1.34	0.81	1.20
Resolution		14.6	6.3	2.1
Theoretical plate number		5189	4934	5043

**Table 14:** Robustness testing results for amiodarone under variations in buffer TEA concentration.

Parameter		Standard (TEA Volume 5.0 mL)	TEA Volume 4.5 mL	TEA Volume 5.5 mL
Recovery (%)	1	99.99	98.73	99.35
	2	100.14	98.59	99.83
	3	100.11	98.50	99.79
Average (%)		100.08	98.61	99.66
Standard deviation (SD)		0.08	0.12	0.27
RSD (%)		0.08	0.12	0.27
Difference (%)		-	1.47	0.42
Retention time (min)		6.61	6.70	6.52
Tailing factor		0.89	0.95	0.97
Symmetry factor		1.34	1.17	1.12
Resolution		14.6	13.8	13.6
Theoretical plate number		5189	5211	5180

**Table 15:** Solution stability results of amiodarone standard and sample solutions over 72 h.

Parameter		Initial	12 h	24 h	48 h	72 h
Standard Peak area	1	5683.162	5640.830	5635.859	5519.935	5451.775
	2	5680.443	5629.122	5627.855	5526.779	5480.521
	3	5681.195	5616.914	5658.175	5504.308	5446.549
	Average	5681.600	5628.955	5640.630	5517.007	5459.615
Sample Peak area	1	5672.978	5595.115	5626.865	5582.120	5546.575
	2	5670.865	5610.450	5628.418	5591.904	5543.336
	3	5649.230	5598.185	5648.904	5585.957	5544.622
	Average	5664.358	5601.250	5634.729	5586.660	5544.844
Recovery (%)	1	99.85	99.40	99.76	101.18	101.59
	2	99.81	99.67	99.78	101.36	101.53
	3	99.43	99.45	100.15	101.25	101.56
Mean recovery (%)		99.70	99.51	99.90	101.26	101.56
Standard deviation (SD)		0.23	0.14	0.22	0.09	0.03
RSD (%)		0.23	0.14	0.22	0.09	0.03
Difference (%)		-	0.19	0.20	1.57	1.87

**Table 16:** Standard concentrations and linearity levels for amiodarone dissolution analysis.

The Sample Concentration Level (%)	Stock Solution (mL)	Total Volume (mL)	Concentration (µg/mL)
20	2	100	44.0
50	5	100	110.0
80	8	100	176.0
100	10	100	220.0
120	12	100	264.0

**Table 17:** Target concentration levels for the preparation of accuracy samples.

The Sample Concentration Level (%)	Stock Solution (mL)	Placebo Stock Solution (mL)	Total Volume (mL)	Concentration (µg/mL)
80	8	11	100	176.0
100	10	11	100	220.0
120	12	11	100	264.0

## Data Availability

The original contributions presented in the study are included in the article and Supplementary Material, further inquiries can be directed to the corresponding authors.

## Supplementary Materials

The following supporting information can be downloaded at: https://www.mdpi.com/article/10.3390/ph19081255/s1, Figure S1: Comparison of HPLC chromatograms of amiodarone using different organic solvents in the mobile phase. (A) Acetonitrile:Buffer (72:28, *v*/*v*) and (B) Methanol:Buffer (72:28, *v*/*v*).

## Abbreviations

The following abbreviations are used in this manuscript:

USP	United States Pharmacopeia
QC	quality control
AQbD	analytical quality by design
ICH	International Council for Harmonisation
ATP	analytical target profile
CAAs	critical analytical attributes
PHA	preliminary hazard analysis
FMEA	failure mode and effects analysis
RP-HPLC	reversed-phase high-performance liquid chromatography
LOD	limit of detection
LOQ	limit of quantitation
RSD	relative standard deviation
S	severity
O	occurrence
D	detectability
RPN	risk priority number
CMPs	critical method parameters
SD	standard deviation
σ	standard deviation of the response
*S*	the slope of the calibration curve
TEA	triethylamine
RH	relative humidity
API	active pharmaceutical ingredient
UV-Vis	ultraviolet–visible
RC	regenerated cellulose
