# Supplementary material for: AQbD-Driven RP-HPLC Method for Rapid Dissolution Monitoring of Amiodarone Hydrochloride Tablets Without Sample Dilution
Source: Pharmaceuticals (Basel). 2026 Aug 9;19(8):1255. doi: 10.3390/ph19081255 (PMC13516048; doi:10.3390/ph19081255)
Supplement: Supplementary file 1 [file pharmaceuticals-19-01255-s001.zip › pharmaceuticals-4450521-supplementary.pdf]

(A)

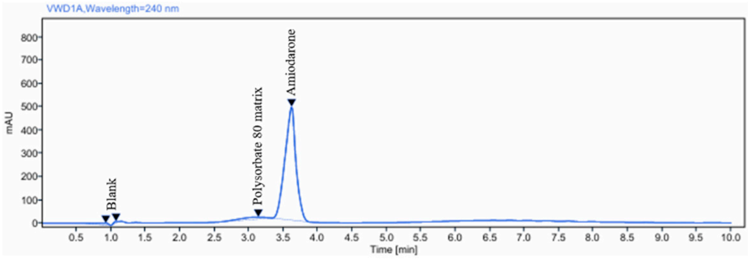

Signal: VWD1A,Wavelength=240 nm

RT [min]	Name	Area	Height	Peak Tail Factor	Peak Symmetry (classic)	Peak Resolution USP	Peak Theoretical Plates USP
0.92		97.082	8.008	0.58980	3.51851		132.73381
1.07		27.513	6.934	0.98761	2.57261	0.72496	2077.42349
3.14		239.272	8.272	0.85867	3.44179	5.54508	371.25890
3.62	Amiodarone	5442.659	487.031	0.88631	1.40156	1.00360	2248.93959

(B)

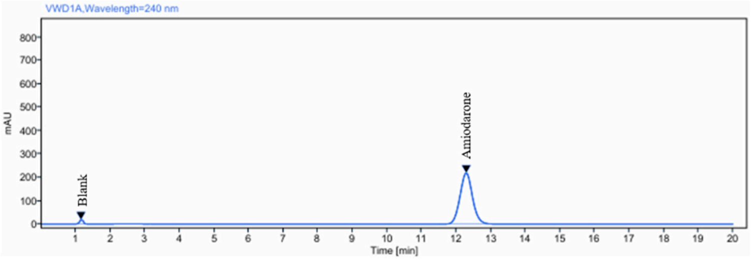

Signal: VWD1A,Wavelength=240 nm

RT [min]	Name	Area	Height	Peak Tail Factor	Peak Symmetry (classic)	Peak Resolution USP	Peak Theoretical Plates USP
1.15		155.782	19.103	1.08313	0.68055		593.13786
12.29	Amiodarone	5511.718	218.832	1.06793	0.95534	26.12982	5477.71585

Figure S1. Comparison of HPLC chromatograms of amiodarone using different organic solvents in the mobile phase. (A) Acetonitrile:Buffer (72:28, v/v) and (B) Methanol:Buffer (72:28, v/v).
